# Similar mutation rates but different mutation spectra in moderate and extremely halophilic archaea

**DOI:** 10.1093/g3journal/jkac303

**Published:** 2022-12-15

**Authors:** Sibel Kucukyildirim, Huseyin Ozgur Ozdemirel, Michael Lynch

**Affiliations:** Department of Biology, Hacettepe University, Ankara 06800, Turkey; Department of Biology, Hacettepe University, Ankara 06800, Turkey; Biodesign Center for Mechanisms of Evolution, Arizona State University, Tempe, AZ 85287, USA

**Keywords:** haloarchaea, *Halobacterium salinarum*, mutation accumulation, mutation rate

## Abstract

Archaea are a major part of Earth’s microbiota and extremely diverse. Yet, we know very little about the process of mutation that drives such diversification. To expand beyond previous work with the moderate halophilic archaeal species *Haloferax volcanii*, we performed a mutation-accumulation experiment followed by whole-genome sequencing in the extremely halophilic archaeon *Halobacterium salinarum.* Although *Hfx. volcanii* and *Hbt. salinarum* have different salt requirements, both species have highly polyploid genomes and similar GC content. We accumulated mutations for an average of 1250 generations in 67 mutation accumulation lines of *Hbt. salinarum,* and revealed 84 single-base substitutions and 10 insertion-deletion mutations. The estimated base-substitution mutation rate of 3.99 × 10^−10^ per site per generation or 1.0 × 10^−3^ per genome per generation in *Hbt. salinarum* is similar to that reported for *Hfx. volcanii* (1.2 × 10^−3^ per genome per generation), but the genome-wide insertion-deletion rate and spectrum of mutations are somewhat dissimilar in these archaeal species. The spectra of spontaneous mutations were AT biased in both archaea, but they differed in significant ways that may be related to differences in the fidelity of DNA replication/repair mechanisms or a simple result of the different salt concentrations.

## Introduction

Mutation is a fundamental component of the evolutionary process. Despite the detailed recent work with bacteria and eukaryotic species (Keightley *et al.* 2009; Ness *et al.* 2015; Lynch *et al.* 2016; Krasovec *et al.* 2017, 2019; Long *et al.* 2018; Pan *et al.* 2021, 2022), little work has been done on the archaea. Thus, to understand variation in genome-wide mutational features across the Tree of Life, it is necessary to expand investigations to include members of this domain, as direct estimates of mutation rate and spectrum are very limited (Kucukyildirim *et al.* 2020; Gu *et al*. 2021). A direct estimate of the genome-wide rate and spectrum of spontaneous mutations can be obtained by mutation accumulation (MA) techniques combined with whole-genome sequencing (WGS). Under this protocol, repeatedly bottlenecking a large number of parallel lineages through single colony/individual transfers minimizes the efficacy of selection by maximizing the power of genetic drift, enabling all but extremely deleterious mutations to accumulate in an effectively neutral manner (Kibota and Lynch 1996; Lynch *et al.* 2008; Keightley and Halligan 2009; Lynch *et al.* 2016).

*Halobacterium salinarum* is an obligate halophilic archaeal species adapted to extremely high salinity—10× that of seawater. Correspondingly, this particular species contains high concentrations of salts intracellularly, and thus has unique molecular characteristics such as metabolic processes operating at saturating salinities. *Halobacterum salinarum* is also able to survive under some other stress conditions such as desiccation and ionizing radiation (Kottemann *et al.* 2005; Kish *et al.* 2009), UV radiation (Shahmohammadi *et al.* 1997; Asgarani *et al.* 1999; Jones and Baxter 2017), and arsenic exposure (Wang *et al.* 2004). Therefore, *Hbt. salinarum* is considered as a model organism for astrobiology (DasSarma 2006; Fendrihan *et al.* 2009; Kunka *et al.* 2020).

The *Hbt. salinarum* NRC-1 genome consists of 2 components: a ∼2 Mb GC-rich (68%) main chromosome and 2 mini-chromosomes about 370 and 190 kb in size (58 and 59% GC content, respectively; Ng 2000). Like many Euryarchaeal species, *Hbt. salinarum* contains more than one copy of the genome per cell—while genetically haploid—with the number of copies variable at different stages of growth (Breuert *et al.* 2006). Our recent study conducted with the polyploid archaeal species *Hfx. volcanii* (65.6% GC content) showed that the per site per generation mutation rate in this organism is similar to that in mesothermophilic bacteria (Kucukyildirim *et al.* 2020). Both *Hbt. salinarum* and *Hfx. volcanii* belong to the class Halobacteria, but diverged ∼600 million years ago (Dennis and Shimmin 1997), although still having similar genomic organization and GC content. Expanding our previous work to *Hbt. salinarum* will improve our understanding of variation in the mutation rate and spectrum of archaea. To this end, we have conducted a mutation-accumulation experiment on *Hbt. salinarum* and compared the mutational profile with that of *Hfx. volcanii*.

## Materials and methods

### Strains, medium, and mutation accumulation

*Halobacterium salinarum* NRC-1 strain was ordered from American Type Culture Collection (ATCC 700922) and recommended growth medium by ATCC [*Halobacterium* medium 2185: 250 g/l NaCl, 20 g/l Mg_2_SO_4_.7H_2_O, 3 g/l trisodium citrate, 2 g/l KCl, 5 g/l tyrptone, 3 g/l yeast extract, and 20 g/l agar supplemented with 0.1 ml/l trace metal solution (6.6 g/l ZnSO_4_.7H_2_O, 1.7 g/l MnSO_4_.7H_2_O, 4.1 g/l Fe(NH_4_)_2_SO_4_.6H_2_O, 0.7 g/l CuSO_4_.5H_2_O)] were used for MA line transfers. Eighty MA lines were established from a single progenitor colony. Single colonies were transferred by re-streaking onto fresh ATCC *Halobacterium* medium 2185 plates at 42°C every week. The number of cell divisions between 2 consecutive transfers was estimated based on colony-forming units (CFUs): Every month single colonies from 10 randomly selected MA lines were transferred to a sterile tube with basal salt solution (medium without carbon source), vortexed, serially diluted and plated to count CFU. After incubation at 42°C (7 days), CFUs were counted and averaged. The number of generations (*n*) was calculated by *n* = log_2_(CFU). The total number of cell divisions of each MA line is the product of the mean (25.1) of all cell division estimates and the total transfer number for each line. On average, each MA line experienced 50 transfers.

*Haloferax volcanii* data used in this work derived from a previous study by Kucukyildirim *et al.* (2020), involving a large-scale MA experiment using ATCC 29605 strain of *Hfx. volcanii* carried for ∼3,000 generations. In brief, *Hfx. volcanii* MA lines were cultivated in ATCC-recommended *Halobacterium* medium 974 plates that contain 0.5× less NaCl compared with the *Halobacterium* medium 2185. The data of the surviving 54 *Hfx. volcanii* MA lines were used in this work to compare the mutation rate and spectrum (NCBI Bioproject No.: PRJNA386190).

### DNA extraction, whole-genome sequencing, and data analyses

DNA was extracted from the surviving 67 MA lines using bacterial DNA extraction kit (AMBRD Laboratories, Turkey). Library construction for DNBseq sequencing platform and WGS were done by Beijing Genomics Institution (BGI-Hong Kong) with 2 × 100 bp run. Across the 67 samples, an average depth of >200× coverage was achieved and, on average, 96.8% of genomic positions were covered. Computational analyses were performed by using Carbonate high-performance computing cluster at Indiana University. Removing of adapters and trimming of low-quality reads were performed by Cutadapt ver1.9.1 (Martin 2011). Then, we applied the same data analysis pipeline that used in the *Hfx. volcanii* study to ensure consistency. In brief, only paired reads were mapped to the *Hbt. salinarum* NRC-1 reference genome (NCBI accession numbers: NC_002607.1, NC_002608.1, and NC_001869.1), using BWA mem, version 0.7.12 (Li and Durbin 2009). Duplicate reads were removed using Picard-tools-1.141, and read mapping around indels were realigned using GATK 3.6, before performing variant discovery with standard hard filtering parameters (except Phred-scaled quality score QUAL > 100 and MQ > 59 for both variant and nonvariant sites; ploidy setting: diploid higher ploidy did not change mutation detection; McKenna *et al.* 2010; DePristo *et al.* 2011). Base substitutions and small indels were called using HaplotypeCaller in GATK. In order to call a variant, a minimum of 10 reads was needed and ≥99% of reads in a line were required to call the line-specific consensus nucleotide at a candidate site; a ∼1% cutoff was set to allow for aberrant reads originating from sequencing errors, contamination of pure indexes during library construction or barcode degeneracy during sequence demultiplexing. We then followed Kucukyildirim *et al.* (2020) for calculations. Statistics were conducted by using R 3.3.2 (R Development Core Team 2015). All mutation sites were visually confirmed with the Integrative Genomics Viewer (IGV 2.3.5; Thorvaldsdottir *et al.* 2013).

## Results

To resolve the genome-wide spontaneous mutation rate and spectrum in *Hbt. salinarum* NRC-1 strain, 80 MA lines were established from a single progenitor colony. Each MA line was transferred every week for 15 months, with 67 lines surviving to the end of the MA. During MA, each line experienced ≥46 single-cell bottlenecks, and on average 1267 cell divisions (Supplementary Table 1), allowing mutations to accumulate across the whole genome in a nearly neutral fashion (Kibota and Lynch 1996; Halligan and Keightley 2009; Lynch *et al.* 2016; Long *et al.* 2018).

### Base-substitution mutation rate and spectrum

**Fig. 1.**
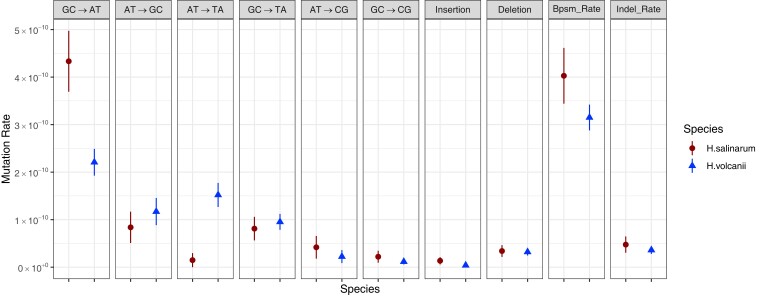
The spectrum of base-pair substitution mutation (bpsm) and indel rates observed for *Hbt. salinarum* and *Hfx. volcanii* MA lines. The circles (*Hbt. salinarum*) and triangles (*Hfx. volcanii*) represent mean base-substitution and indel rates for each BPS type normalized by the nucleotide base composition of the 2 genomes; bars indicate 95% confidence intervals of the mean.

We detected 84 base substitutions, yielding a mutation rate of 3.99 × 10^−10^ (SE = 0.59 × 10^−10^) per site per generation or 1.0 × 10^−3^ per genome per generation (Supplementary Tables 1). This observation is relatively consistent with most of the previously reported mesothermophilic prokaryotic species, including *Hfx. volcanii*, although the *Hbt. salinarum* (per site per generation) base-substitution mutation rate is slightly higher than observed in *Hfx. volcanii* (Table 1). In addition, our estimate is similar to the previously reported 1.7 × 10^−3^ per genome per generation mutation rate in *Hbt. salinarum* based on a reporter-construct estimate (Busch and Diruggiero 2010). Regarding the spectrum of base substitutions between these two haloarchaeal species, we found a significant difference (*χ*² = 22.46, d.f. = 5, *P* < 1 × 10^−3^; Fig. 1). The base-substitution mutations in *Hbt. salinarum* are noticeably dominated by G/C→A/T transitions, rendering the transition:transversion (Ts/Tv) ratio (4.71) higher than in *Hfx. volcanii* (1.42) and in most bacterial species with resolved mutation spectra (Long *et al.* 2018).

**Table 1.** Mutation patterns in *Hbt. salinarum* and *Hfx. volcanii* mutation accumulation lines.

	*Hbt. salinarum*	*Hfx. volcanii*
No. of sites surveyed	2,489,236	3,003,077
Average number of generations	1,267	3,274
No. of base substitutions	84	167
No. of indels	10	19
Transitions/transversions	4.71	1.42
No. of coding substitutions	63	120
No. of noncoding substitutions	21	47
Overall base-substitution mutation rate (× 10^−10^)	3.99	3.15
Overall insertion-deletion rate (×10^−11^)	4.75	3.58

The mutation rate in the AT direction µ_G/C→A/T_ (including G:C→A:T transitions and G:C→T:A transversions) is 5.09 × 10^−10^ (95% Poisson confidence interval: 3.98–6.42 × 10^−10^), while the mutation rate in the GC direction µ_A/T→G/C_ (A:T→G:C transitions and A:T→C:G transversions) is 1.26 × 10^−10^ (0.58–2.40 × 10^−10^; Supplementary Table 1). Given the observed mutation rates in *Hbt. salinarum*, the expected GC content at mutation equilibrium is ∼20% (SEM = 0.038), much lower than the actual genome GC content of 65.9%. This type of bias has been observed in most other species, implying that genome-wide GC content is generally driven by other evolutionary forces such as natural selection (Long *et al.* 2018).

Across all sequenced *Hbt. salinarum* MA lines, there were 73 base-substitution mutations in the main chromosome, 1 on pNRC100, and 10 on pNRC200 (Supplementary Tables 2–4). Regarding the different length and GC content of the main and mini-chromosomes, we examined overall base-substitution mutation rates of each chromosome with a *χ*² test and revealed no significant difference (*χ*² = 5.5, d.f. = 2, *P* = 0.06), and this result is consistent with previous work with *Hfx. volcanii* (Kucukyildirim *et al.* 2020). But, it is different from the prior observation suggesting that mini-chromosomes have higher mutation frequencies than the main chromosome in *Hbt. salinarum* (Kunka *et al.* 2020), which may be caused by different experimental approaches used.

Using the annotated *Hbt. salinarum* genome, we parsed the functional context of all base substitutions (Supplementary Table 5). Across the 67 sequenced MA lines, 63 of the 84 (75%) substitutions are located in coding regions, while the remaining 21 are found at noncoding sites. To test whether selection may have influenced the distribution of mutations in this experiment, we estimated the base-substitution mutation rate in coding regions (3.56 × 10^–10^ per nucleotide site per generation; 95% confidence intervals: 2.74 × 10^–10^, 4.56 × 10^–10^); and noncoding regions (5.06 × 10^–10^; 95% confidence intervals: 3.13 × 10^–10^, 7.73 × 10^–10^), and found that they are not significantly different (Fisher’s exact test, *P* > 0.05).

### Rate and spectrum of insertions and deletions (indels)

**Fig. 2.**
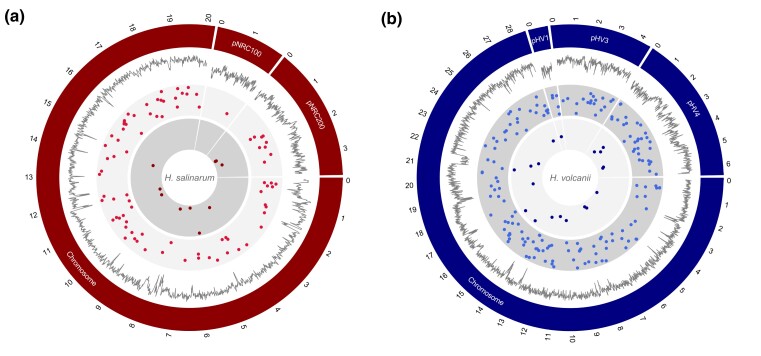
The distribution of the base-pair substitution (bps) and insertion-deletion (indel) mutations in *Hbt. salinarum* (a) and *Hfx. volcanii* (b) MA lines. From the outer track to inner track scaled to genome size (bin size: 100 kb), G/C content (bin size: 1 kb), position of each base substitution in each MA line and position of each indel in each MA line (*Hfx. volcanii* MA lines do not have any mutation on pHV2).

Across all 67 MA lines, we detected 7 small deletions and 3 insertions 1–30 bp in length (Supplementary Tables 1 and 6), yielding an indel rate of 4.75 × 10^−11^ (SE = 1.71 × 10^−11^) per site per generation. This indel rate is ∼1.3× higher than that of *Hfx. volcanii*, and the spectra of indels of both archaeal species are biased toward deletions (Table 1), consistent with prior observations obtained from MA studies (Sung *et al.* 2016). Indels were also more frequent on the main chromosome in both *Hbt. salinarum* and *Hfx. volcanii* (Fig. 2). This slight difference observed between the 2 haloarchaeal species in the genome-wide insertion-deletion rate may be a result of differences in the fidelity of DNA repair mechanisms or a simple result of the environment (high salt concentrations).

## Discussion

Mutation-accumulation experiments combined with WGS reveal an unbiased and direct view of genome-wide spontaneous mutation rates and spectra of a second halophilic member of the archaea. Although the two archaeal species in this study are widely considered to be closely related, they are estimated to have diverged from each other about 600 million years ago. With this work, we determined similar per genome per generation spontaneous mutation rates in *Hfx. volcanii* and *Hbt. salinarum*, with both being consistent with previous findings in mesothermophilic, aerobic prokaryotes. However, we also found that some mutational characteristics are somewhat dissimilar in these archaeal species (e.g. the genome-wide insertion-deletion rate and spectrum of mutations), suggesting a differentiated fidelity of DNA replication/repair enzymes. Archaea have exceptional resistance to harsh environmental conditions (such as high or low temperatures and salinity), which has been associated with efficient DNA repair and detoxification systems (Asgarani *et al.* 1999; Kottemann *et al.* 2005; Rampelotto 2013). Moreover, as previously suggested, it is possible that some DNA replication/repair enzymes might gain novel roles in archaea (Zhang *et al.* 2021), and that highly polyploid species like haloarchaea might increase demand on replication/repair proteins (Pérez-Arnaiz et al. 2020). Consistently, *Hfx. volcanii* encodes 4 uracil-DNA-glycosylase (HVO_0231, HVO_1038, HVO_2792, HVO_0444), and 2 adenine-DNA-glycosylase (MutY) proteins (HVO_2896 and HVO_2834), while *Hbt. salinarum* has 3 uracil-DNA-glycosylase (VNG_2082G, VNG_0707C, and VNG_1228C) and 1 MutY genes (VNG_1520G). The existence of additional base-excision repair enzymes in *Hfx. volcanii* might improve to the prevention of mutations due to uracil and 8-oxoguanine or adenine/guanine mismatches. But the functionality of these enzymes in base-excision repair remains to be elucidated. In addition, both haloarchaeal species analyzed in this work have 2 active mismatch repair systems (the canonical MutL-MutS pathway and the noncanonical NucS pathway; Castaneda-Garcia et al. 2017; Pérez-Arnaiz et al. 2020). While *Hfx. volcanii* encodes 4 MutS (HVO_1940, HVO_0552, HVO_0191, and HVO_1354), 2 MutL (HVO_1939 and HVO_0551) and 1 NucS protein (HVO_0486), *Hbt. salinarum* has 3 MutS (VNG_0172G, VNG_0163G, and VNG_2270G), 1 MutL (VNG_0159G), and 2 nucS genes (VNG_1986G and VNG_0171G). Previous observations were dissimilar about the possible roles of the canonical MutL-MutS pathway in *Hbt. salinarum* and *Hfx. volcanii*. Whereas, in *Hbt. salinarum*, inactivation of mutL or mutS resulted in no hypermutability (Busch and Diruggiero 2010), the deletion of the same genes led to an increase in the spontaneous mutation rate of *Hfx. volcanii* (Pérez-Arnaiz et al. 2020). Thus, the possible interaction between both mismatch repair pathways and their contribution to genome-wide mutation rate and spectrum remains to be clarified. Our observations of similar mutation rates in the face of an altered mutation spectrum as observed in the two haloarchaeal species is consistent with the idea that selection operates on the total mutation rate, with the mutation spectrum being able to drift conditional on the maintenance of the expected overall rate (Lynch *et al.* 2016; Long *et al.* 2018).

## Supplementary Material

jkac303_Supplementary_Data

## Data Availability

Raw sequences are available at the Sequence Read Archive at NCBI (Bioproject no.: PRJNA844510). Supplemental material available at G3 online.
